# Detailed Clinical, Ophthalmic, and Genetic Characterization of *MYO7A*-Associated Usher Syndrome

**DOI:** 10.1167/iovs.66.4.60

**Published:** 2025-04-21

**Authors:** Juan C. Romo-Aguas, Thales A. C. de Guimarães, Angelos Kalitzeos, Nancy Aychoua, Chrysanthi Tsika, Anthony G. Robson, Yu Fujinami-Yokokawa, Kaoru Fujinami, Omar A. Mahroo, Andrew R. Webster, Michel Michaelides

**Affiliations:** 1 Moorfields Eye Hospital, London, United Kingdom; 2 UCL Institute of Ophthalmology, University College London, London, United Kingdom; 3 Laboratory of Visual Physiology, Division of Vision Research, National Institute of Sensory Organs, NHO Tokyo Medical Center, Meguro-ku, Tokyo, Japan

**Keywords:** MYO7A, usher syndrome, retinitis pigmentosa, natural history, genetics

## Abstract

**Purpose:**

To analyze the clinical spectrum and natural history of *MYO7A*-associated Usher syndrome type I (USH1)

**Methods:**

Patients with molecularly confirmed *MYO7A*-associated USH1 in a single tertiary referral center. Data was extracted from physical and electronic case notes, including imaging and electrophysiology. Genetic results were reviewed, and the detected variants were assessed. Main outcome measures were clinical findings, qualitative and quantitative analysis of retinal imaging, and electrophysiology.

**Results:**

Eighty patients were identified and evaluated longitudinally. The mean age (±SD) of onset of symptoms was 12.0 ± 5.8 years of age, and a mean follow-up time of 16.2 years. BCVA was 0.4 ± 0.5 LogMAR at baseline, and 0.7 ± 0.8 LogMAR at the last visit for both eyes. The change in BCVA over time was 0.02 LogMAR per year. A hyperautofluorescent (hyperAF) ring was present in 51% of the patients. The mean ellipsoid zone width (EZW) at baseline was 2568.2 ± 1528.9 µm OD and 2527.9 ± 1609.3 µm OS, which decreased to 2012.3 ± 1705.1 µm OD and 1806.3 ± 1647.1 µm OS at last visit. Electrophysiology revealed rod and cone dysfunction with relative or complete sparing of macular function. There were statistically significant changes in BCVA, EZW, and hyperAF ring between baseline and follow-up. Genetic analysis identified 83 variants in *MYO7A*, including 18 novel variants.

**Conclusions:**

Longitudinal analysis shows that the majority of patients retain central visual function and structure until the fifth decade of life, which informs advice on prognosis and the window for therapeutic intervention.

Usher syndrome (USH) refers to a heterogeneous group of autosomal recessive conditions characterized by progressive visual loss caused by retinitis pigmentosa (RP) and varying degrees of congenital sensorineural hearing loss (SNHL) and vestibular symptoms.^1^ The prevalence of USH in the general population is between three to 17 per 100,000 people, and it is the most common genetic cause of combined dual sensory impairment.^2^^–^^4^ Classically, three USH subtypes have been described based on the onset, progression and severity of the clinical manifestations, although a fourth atypical form of USH has also been reported.^5^^–^^7^ Type 1 (USH1) is considered the most disabling because of its presentation of congenital profound SNHL, vestibular dysfunction, and RP onset within the first two decades of life.^8^

There are currently five genes associated with USH1, and the most prevalent is *MYO7A* (OMIM *276903, also known as DFNB2, USH1B) accounting for 29% to 50% of cases.^9^^,^^10^ It encodes the protein myosin VIIa and is expressed in the retina and the inner ear. Two isoforms have been found in the retina, which differ from each other because of alternate splicing of exon 35.^11^ They are expressed in cone and rod photoreceptors and in the retinal pigment epithelium, where the encoded protein is involved in opsin transport through the connecting cilium and the movement of melanosomes and phagosomes.^12^^–^^14^

Herein, we provide a detailed characterization of the phenotypic and genetic features of a large cohort of 80 individuals from a single institution with *MYO7A* associated USH1. This data is vital for genetic counseling, monitoring, and prognostication, while also providing crucial information to help design prospective natural history studies and clinical trials.

## Material and Methods

This retrospective study adhered to the tenets of the Declaration of Helsinki and received approval from the local ethics committee. Informed consent was obtained from all adult patients, whereas informed consent and assent were obtained from parents and children, respectively, as indicated.

### Patient Identification

Patients with molecularly confirmed *MYO7A*-associated USH1 were identified by reviewing the clinical records and genetics database of Moorfields Eye Hospital (London, UK). All patients harbored two or more *MYO7A* variants and were seen by retina genetics specialists (MM, OM, ARW). Patients with other pathologies or questionable diagnosis were excluded from further analysis.

Relevant patient data was retrieved from the electronic and physical health care records and imaging software systems. The age of disease onset was defined as the age of the first disease-related symptom(s). Snellen visual acuities were recorded and converted to logarithm of the minimum angle of resolution (LogMAR) for descriptive statistics.

For patients who had recorded count fingers vision, hand motion, light perception and no light perception, the following values were recorded: LogMAR 2.10, LogMAR 2.40, LogMAR 2.70, and LogMAR 3.0, respectively.^15^^,^^16^ Patients were categorized using the World Health Organization visual impairment criteria (based on best-corrected visual acuity [BCVA] of the best seeing eye), which defines no or mild visual impairment as BCVA ≤0.48 (6/18), moderate impairment as BCVA >0.48 and ≤1.0 (6/60), severe as BCVA >1.0 and ≤1.3 (3/60), and blindness as BCVA >1.3. Very limited visual field data was available; therefore we classified patients based on BCVA.

### Retinal Imaging

Imaging modalities were obtained under dilated fundus examination with spectral-domain optical coherence tomography (OCT; Heidelberg Spectralis; Heidelberg Engineering, Inc, Heidelberg, Germany), fundus autofluorescence (FAF; Heidelberg Spectralis and Optos PLC, Dunfermline, UK), and ultrawide field fundus color photography (Optos PLC).

Qualitative assessment of color fundus photos and FAF/OCT images was performed using all available data from our site, at baseline and last follow-up. Quantitative analysis was performed in all patients by a single experienced observer (JCRA). Central macular thickness (CMT), ellipsoid zone width (EZW), and outer nuclear layer thickness (ONLT) were recorded. Qualitatively, the presence of cystoid macular edema (CMO), and epiretinal membrane (ERM) was noted.

The EZW was measured using digital callipers (Heidelberg Eye Explorer version 2.6.5.0; Heidelberg Engineering), at a 1 µm:1 µm display with maximum magnification, on the transfoveal horizontal line scan, with the foveal reflex used as an anatomical landmark. The ONLT was calculated as the subfoveal distance between the internal limiting membrane and the external limiting membrane. The area within the hyperautofluorescent (hyperAF) ring at the macula was quantified by tracing the border of the ring manually using the software area tools (Heyex version 2.6.5.0; Heidelberg Engineering). We used 55° × 55° images given the large area of some of the rings that would otherwise not be measurable in the 30° × 30° images. When a double ring was present, the innermost ring was measured. If the ring area was beyond the edge of the FAF image, the scan was excluded. The follow-up mode allowed a more accurate measurement in the longitudinal analysis.

### Genetic Testing

DNA was extracted from whole blood, and genetic testing was performed using panel-based targeted next-generation sequencing, whole exome sequencing, or whole genome sequencing. Where appropriate and available, blood samples were taken from parents or siblings to confirm the segregation of proposed variants. In silico analysis was performed for previously unreported variants. The pathogenicity of each variant was classified according to the guidelines of the American College of Medical Genetics and Genomics.^17^^–^^19^ In silico molecular modeling and conservation analysis are described in Suppelementary Methods S1 and Table S3, respectively.

### Electrophysiology

Nineteen patients (19/80 = 23.8%) underwent full-field and pattern electroretinogram (ffERG; PERG) testing, incorporating the International Society for Clinical Electrophysiology of Vision (ISCEV) Standard methods,^20^^,^^21^ using gold foil corneal recording electrodes. Full-field ERGs, recorded under dark-adapted (DA) and light-adapted (LA) conditions, were used to assess generalized rod and cone system function, and pattern ERG P50 was used as a measure of macular cone system function.^22^ The ERG data was compared with a reference range from a group of healthy subjects (age range 10–79 years).^23^^,^^24^ The amplitudes of the main ffERG and PERG P50 components were plotted as a percentage of the age-matched lower limit (fifth percentile) of the reference range or as a difference from the age-matched upper limit for peak time (95^th^ percentile). In addition, five children aged four, six (*N* = 2), and seven (*N* = 2) years were tested according to a shortened photopic and scotopic ERG protocol^25^ using lower eyelid skin electrodes without mydriasis, using Ganzfeld (*N* = 1; age seven years) or non-Ganzfeld flashes (*N* = 4).

### Statistical Analysis

**Figure 1.**
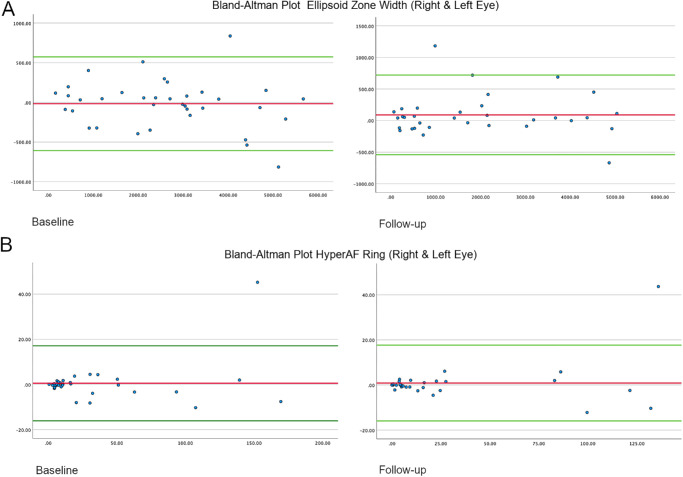
Bland-Altman Plot. The graph shows the distribution of EZW in µm and hyperAF area in mm^2^ of both eyes at baseline and follow-up. The *red lines* represent the mean bias, and the *green lines* represent the upper and lower 95% confidence interval.

**Figure 2.**
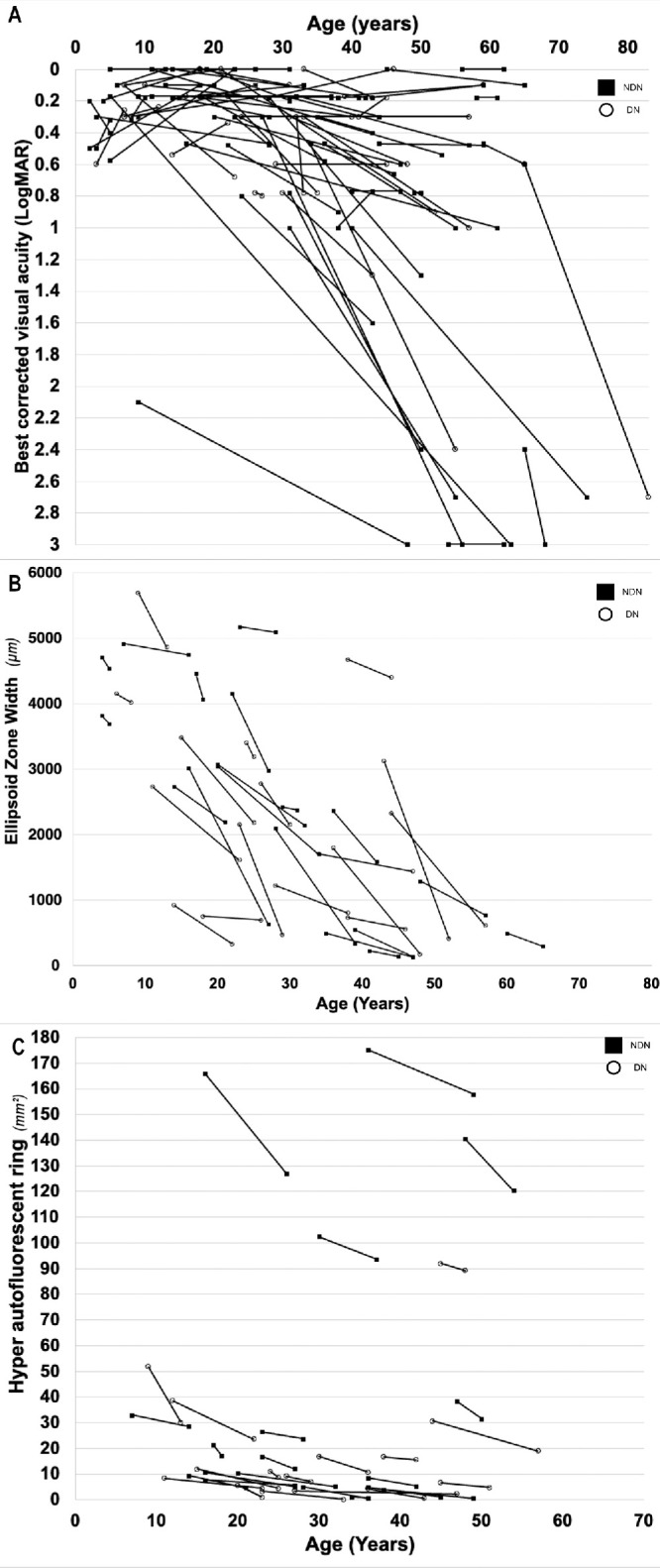
Baseline-follow up values indicating change over time. Plotted from observations of **(A)** BCVA (*n* = 74), **(B)** EZW) (*n* = 35, and **(C)** hyperAF ring area (*n* = 34). Each datapoint pair represents the right eye of a subject across their follow-up time. Datapoints with *hollow circles* represent patients with NDN genotype, and datapoints with *filled circles* represent patients with DN genotype. **(C)** The distinct group of five datapoint pairs extending beyond 80 mm^2^ hyper-AF ring area belong to patients within the mild phenotype.

Statistical Packages for Social Sciences software version 28.0 (IBM, Armonk, NY, USA) was used for the data analysis. Descriptive statistics were generated for continuous variables and categorical variables. Continuous variables were reported as either means with standard deviation or medians with interquartile ranges (IQR) depending on normality testing results. Our threshold of statistical significance was set at *P* ≤ 0.5. Normality test (Kolmogorov-Smirnov) was performed to assess data distribution and Bland-Altman plots were drawn to assess interocular difference for each patient at baseline and follow-up (Fig. 1). When symmetry was established, only the right eye was reported. We measured change over time, defined as the difference in values obtained over two time points (baseline and follow-up), divided by the length of follow-up in years. This was calculated for BCVA, EZW, and area of hyperAF rings. In addition, “baseline-follow up” values were plotted using the two data points in Excel (version 25.004.0109; Microsoft, Inc., Redmond, WA, USA) as shown in Figure 2.^26^^–^^28^ The effect of age and variant severity on baseline was assessed with ANOVA, where subjects were divided into age groups (1) <10 years, (2) 11–20 years, (3) 21–30 years, (4) 31–40 years, and (5) > 40 years. For the assessment of genotype, patients were categorized into two groups based on presence of one null variant (1) non-double null (NDN); or two null variants (2) double null (DN). A *t*-test was done for parametric variables’ assessment and Wilcoxon's signed-rank test for nonparametric variables. Kaplan-Meier survival curves represent the visual acuity loss at different decades.

## Results

### Demographics and Symptoms

Eighty patients from 77 families were identified. Their clinical characteristics are summarized in Table 1. Forty-three (53.8%) patients were male. The mean age (± SD) at the first visit was 25.4 ± 16.7 (range 2–72). All patients (100%) had congenital hearing impairment, and 20 (25%) reported some degree of vestibular symptoms. Thirty-one (38.8%) had cochlear implants, and six (7.5%) patients with less severe hearing loss used hearing aids. The mean age for cochlear implant surgery was 11.7 ± 15.3 (median: 6.0, range 2–56). Consanguinity was reported in 7 families, with parents being first cousins in all cases.

**Table 1.** Clinical Characteristics of Patients With Usher Syndrome Type 1 Associated With *MYO7A*

Characteristics	Patients (*n* = 80)
Families	77
Gender	
Male	43 (53.8%)
Female	37 (46.3%)
Age at first examination (y), mean ± SD	25.4 ± 16.7
Age at last examination (y), mean ± SD	40.8 ± 17.3
Follow-up time (y), mean ± SD	16.2 ± 11.4
Age of onset of visual symptoms (y), mean ± SD	12.0 ± 5.8
Cochlear implant	31 (38.8%)
Age at cochlear implant (y), mean ± SD; median	11.7 ± 15.3; 6.00
Reported first symptom	74 (92.5%)
Night blindness and visual field loss	21 (28.4)
Night blindness	34 (45.9)
Visual field loss	11 (14.9)
Cataract	48 (60%)
Age of cataract (y), mean ± SD	35.0 ± 9.2

The age of the first visual symptoms was recorded for 74 (92.5%) patients at the mean age of 12.0 ± 5.8, with eight (10.8%) patients being asymptomatic. Thirty-four (45.9%) patients reported nyctalopia as their first symptom, 21 (28.4%) patients had combined night vision problems and peripheral vision loss, and 11 (14.9%) reported loss of visual field. At the first visit, 60 (80%) patients had mild or no visual impairment, 14 (17.5%) had moderate visual impairment, and two (2.5%) patients were blind (age 54 and 65).

### BCVA and Fundus Findings

**Figure 3.**
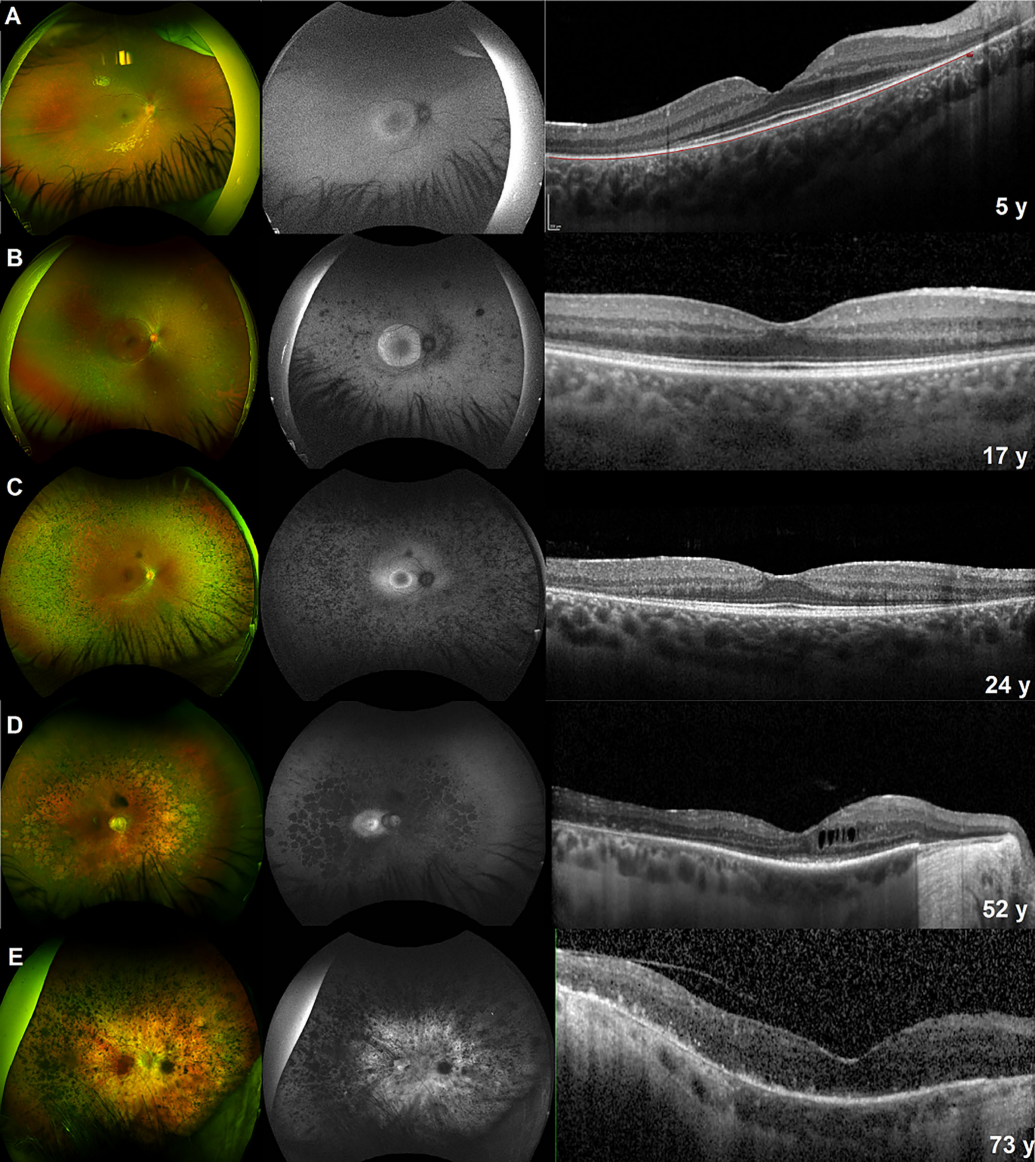
Multimodal imaging of five patients from different age groups. Imaging findings in patients with *MYO7A*-associated Usher syndrome type 2. Here we show a composition of pseudocolor wide-field fundus photography, fundus autofluorescence, and optical coherence tomography from five unrelated patients of different groups of age. Patients MYO7A 018 **(A)**, MYO7A 002 **(B)**, MYO7A 003 **(C)**, MYO7A 024 **(D)**, and MYO7A 056 **(E)**, respectively.

**Figure 4.**
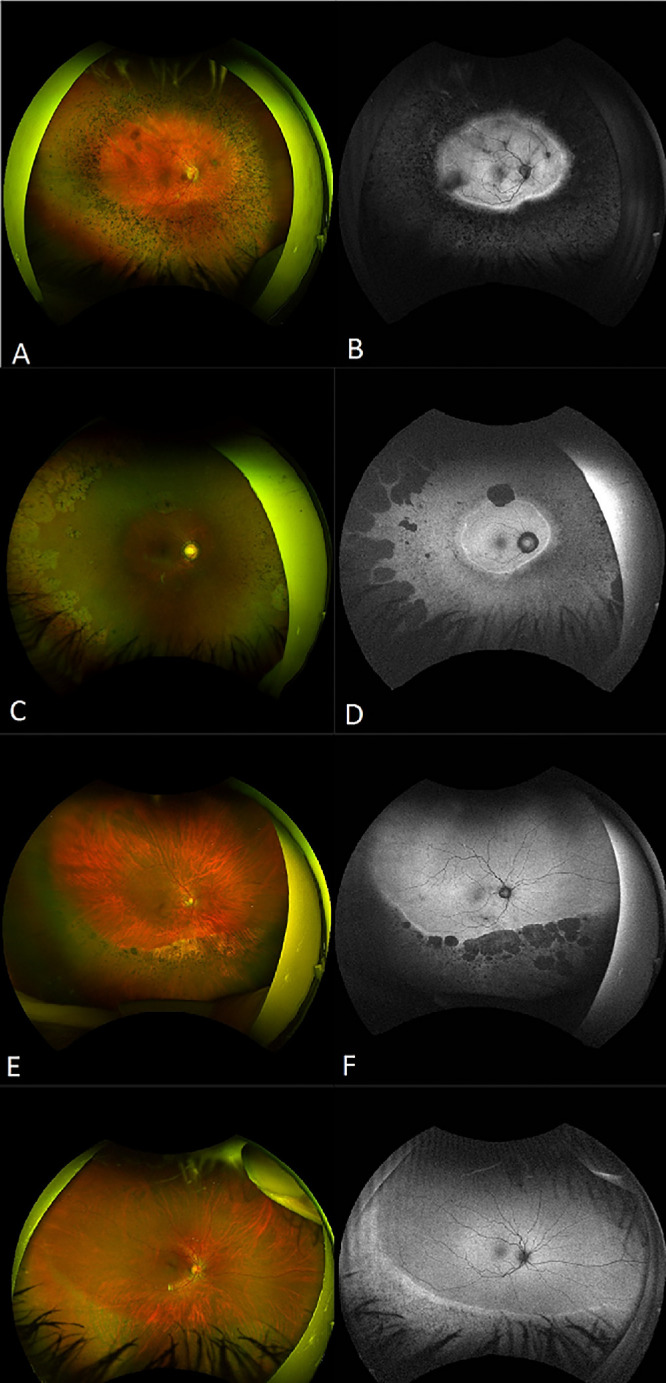
Multimodal imaging of four patients with a milder phenotype. Pseudocolor wide-field fundus photography, fundus autofluorescence and optical coherence tomography, of two patients with mild generalized retinitis pigmentosa and two patients with sector retinitis pigmentosa, **(A, B)** MYO7A 066 [age 21], MYO7A 046 **(C, D)** [age 39], MYO7A 066 **(E, F)** [age 45], and MYO7A 040 **(G, H)** [age 56].

At baseline, the median BCVA was 0.3 LogMAR (IQR 0.17–0.5 LogMAR). Forty-eight (60%) patients developed a cataract at the mean age of 35.0 ± 9.2 years. Color fundus photographs were available for 70 (87.5%) patients. Twenty-eight (43.75%) patients had mild to moderate bone-spicule-like (BSL) pigment deposits in the periphery, 27 (42.18%) had dense BSL deposits, and nine (14.06%) had no pigment (Fig. 3). A milder RP phenotype was found in 6 (8.5%) patients with a wider preserved retina outside the macula, and 6 (8.5%) patients had sector RP (Fig. 4). Optic nerve head drusen were present in nine (12.5%) patients.

### Retinal Imaging

#### Interocular Symmetry

Bland-Altman analysis of the EZW and hyperAF ring area showed a good interocular agreement at baseline and follow-up. The mean bias (OD − OS) for EZW at baseline and follow-up was −16.6 µm (SD 301.5 µm) and 91.3 µm (SD 321.7 µm), and 95% of differences falling between −607.5 and 574.4 µm, and −539.3 µm and 721.9 µm, respectively. Regarding the hyperAF ring area, the mean bias (OD − OS) at baseline and follow-up was 0.53 mm^2^ (SD 8.47 mm^2^) and 0.87 mm^2^ (SD 8.58 mm^2^), and 95% of differences falling between −16.1 and 17.2 mm^2^, and −15.9 and 17.7 mm^2^, respectively.

### FAF Analysis

Seventy-one (88.8%) patients had FAF imaging available, and 36 (51%) patients had bilateral hyperAF rings. The mean area of the hyperAF ring at baseline was 33.5 ± 45.8 mm^2^, which was not significantly associated with age (*P* = 0.35).

### OCT

Seventy-two (90%) patients had OCT scans available. Nine (12.5%) patients had a unilateral ERM, seven (9.7%) had bilateral ERM, and one (1.3%) patient had a unilateral full-thickness macular hole. Thirteen (18.1%) patients had bilateral CMO, and nine (12.5%) patients had unilateral CMO at baseline. These patients were excluded from the quantitative thickness analysis (*n* = 22 eyes excluded). The mean age of patients with CMO was 40.2 ± 15.5 years, and those without was 35.16 ± 18.9 years.

The CMT was 252.5 ± 64.2 µm OD and 257.5 ± 63.8 µm OS. The ONLT was quantifiable in 48 (66.6%) patients with a mean value of 96.7 ± 22.3 µm. EZW was quantifiable in 38 (52%) patients, excluding the patients with mild RP phenotype and sector RP, who had a complete EZW in the macular subfoveal scan (*n* = 12). The mean EZW was 2568.1 ± 1528.9 µm OD and 2527.9 ± 1609.3 µm OS.

### Longitudinal Analysis

**Figure 5.**
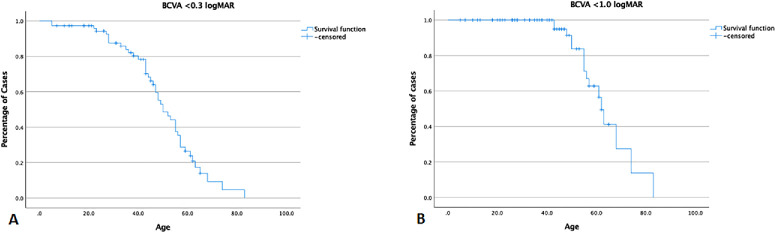
Kaplan-Meier survival analysis showing the percentage of patients with a range of baseline best-corrected visual acuities. **(A)** Percentage of patients with BCVA ≤0.3 LogMAR (6/12) in at least one eye (50% at 53 years of age) and **(B)** the percentage of patients with legal blindness (≥1 LogMAR, 50% at 62 years of age).

The mean follow-up time was 16.2 ± 11.4 years with a mean age at the final visit of 40.8 ± 17.3 years. Figure 2 shows ”baseline-follow-up” values for every patient for whom follow-up was available and was more than year apart, showing the distribution of BCVA, EZW and HyperAF ring area. At the last visit, the median BCVA was 0.4 LogMAR (IQR 0.17–0.8 LogMAR) OD and 0.3 LogMAR (IQR 0.17–0.8) OS. There was a significant difference between the BCVA at baseline and at final follow-up (*P*≤ 0.001). The change over time was 0.02 LogMAR/year. Kaplan-Meier survival analysis showed that at 43 years of age, 75% of the patients had a BCVA of 0.3 LogMAR (6/12) or better and this drops to 50% at 50 years of age. It also showed that at 62 years of age, 50% of the patients had legal blindness based on BCVA ≥1 LogMAR (20/200, 6/60) (Fig. 5).

Thirty-four patients had at least two consecutive FAF images with a hyperAF ring, and the mean follow-up time was 6.3 ± 4.1 years. The baseline mean area of the hyperAF ring was 27.7 ± 45.8 mm^2^ OD and 29.8 ± 44.4 mm^2^ OS. The change over time was 1.2 ± 1.2 mm^2^ per year. There was a statistically significant difference between the area of the hyper AF ring at baseline and the end of follow-up (*P* ≤ 0.001).

The mean follow-up of patients with at least two OCT scans was 6.9 ± 3.9 years. The average CMT at follow-up was 253.7 ± 62.0 µm OD and 260.6 ± 57.6 µm OS. At the end of follow-up, ONLT was measurable in 45 patients with a mean of 91.5 ± 24.1 µm OD and 95.9 ± 23.9 µm OS. Thirty-five patients still had an EZW at the last appointment with a mean of 2012.3 ± 1705.1 µm OD and 1806.3 ± 1647.1 µm OS. The EZW change over time was 25.2 ± 21.6 per year. Differences between baseline and last follow-up were significant for, ONLT (*P* ≤ 0.001) and EZW (*P* ≤ 0.001) (Table 2).

**Table 2.** Longitudinal Assessment of BCVA and Imaging Parameters

Variables	Baseline	Follow-Up	Wilcoxon Signed Rank Test (*P* Value)
BCVA (LogMAR), mean ± SD	*N* = 80	*N* = 74	
OD	0.4 ± 0.5	0.7 ± 0.8	<0.001
OS	0.4 ± 0.5	0.6 ± 0.8	<0.001
CMT (µm) mean ± SD	*N* = 50	*N* = 44	
OD	252.50 ± 64.2	253.7 ± 62.0	0.13
OS	247.46 ± 63.8	260.6 ± 57.6	0.058
EZW (µm), mean ± SD	*N* = 38	*N* = 35	
OD	2568.2 ± 1528.9	2012.3 ± 1705.1	<0.001
OS	2527.9 ± 1609.3	1806.3 ± 1674.1	<0.001
ONLT	*N* = 48	*N* = 45	
OD	96.7 ± 22.3	91.5 ± 24.1	<0.001
OS	98.08	95.9 ± 23.9	0.004
HyperAF ring (mm^2^), mean ± SD	*N* = 36	*N* = 36	
OD	33.5 ± 45.7	27.7 ± 45.8	<0.001
OS	33.1 ± 44.4	29.8 ± 44.4	<0.001

OD, right eye; OS, left eye.

### Electrophysiological Assessment

There was a high degree of interocular ERG symmetry based on amplitudes of the ISCEV Standard DA 0.01, DA 10 ERG a- and b-waves, LA 30Hz ERG and LA 3 (single flash) ERG b-waves (slope = 0.99; *r*^2^ = 0.99; *n* = 19), and LA 30Hz peak times, with a maximum peak time difference between eyes of 2 ms (*n* = 14).

**Figure 6.**
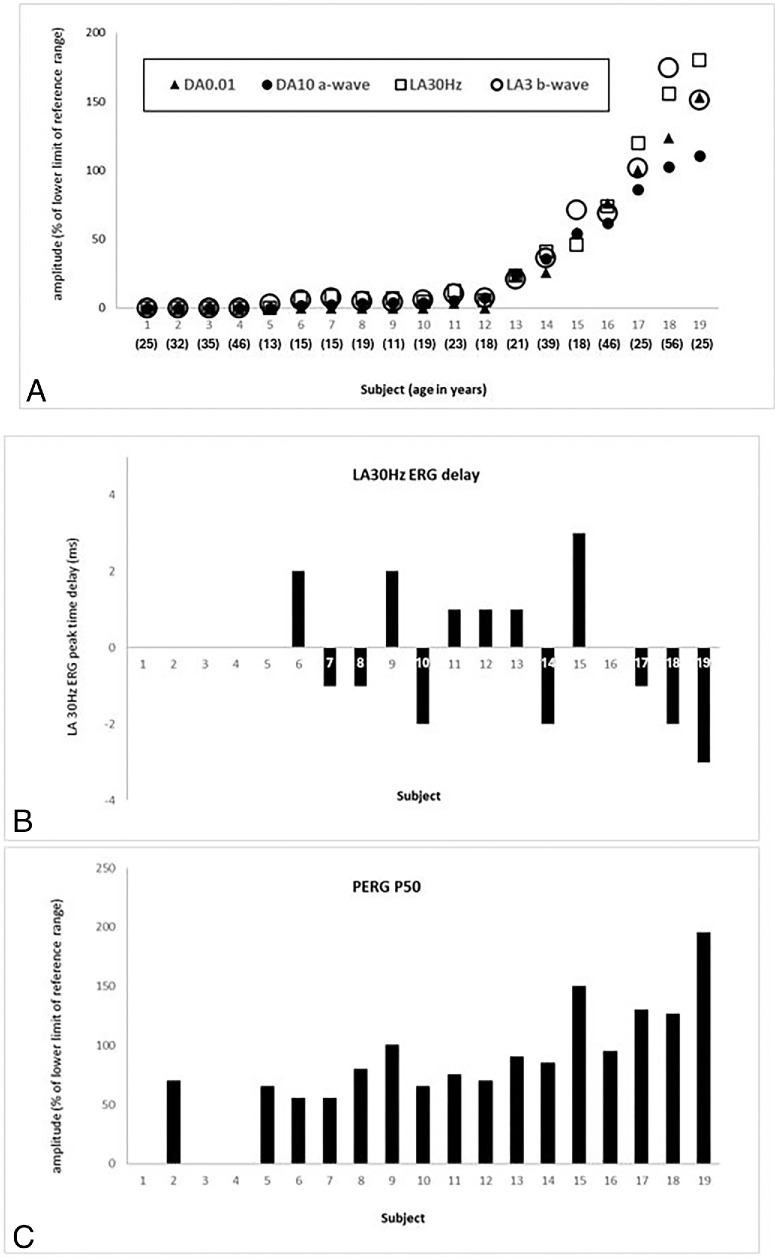
Full-field ERG and PERG P50 findings. Full field and pattern ERG findings in 19 subjects that were tested according to ISCEV standard methods. **(A)** The amplitudes of the DA 0.01 ERG, DA 10 ERG a- wave, LA 30 Hz ERG and LA 3 ERG b-wave are plotted as a percentage of the age-matched 5th percentile of the (“normal”) reference range, with values arranged in ascending order of DA10 ERG a-wave amplitude for clarity. **(B)** The LA 30 Hz peak times are plotted as a difference from the age-matched 95th percentile of the reference range (x-axis indicates 0 ms delay). **(C)** The ISCEV Standard PERG P50 amplitudes of the same patients, expressed as a percentage of the lower limit of the (“normal”) reference range. Subjects 5 and 9 had double null *MYO7A* variants. All electrophysiological recordings showed a high degree of interocular symmetry and are shown for right eyes only.

The ISCEV Standard ERG and PERG P50 findings and patient ages at the time of testing are summarized in Figure 6^7^. The ffERG phenotypes ranged from undetectable DA and LA ERGs (*n* = 4) to responses that had amplitudes within the reference range to all flash stimuli (*n* = 2). In those with detectable but abnormal ERGs, severe reductions (less than 20% of the lower reference limits) were seen in eight individuals, and more moderate reductions were seen in an additional five cases, with similar involvement or rod and cone photoreceptors in most, including one case with evidence of mild and selective loss of rod photoreceptor function. It is highlighted that the two cases with ERG amplitudes that fell within the reference range (including the twin of the case with mildest DA10 ERG a-wave reduction) had proportionately smaller DA10 ERG a-waves compared with their LA ERGs, suggestive of a mild loss of rod function. LA 30Hz ERG peak times were normal in seven of 14, with a detectable response and showed marginal-mild delay in six others (by 1-3 ms).

**Figure 7.**
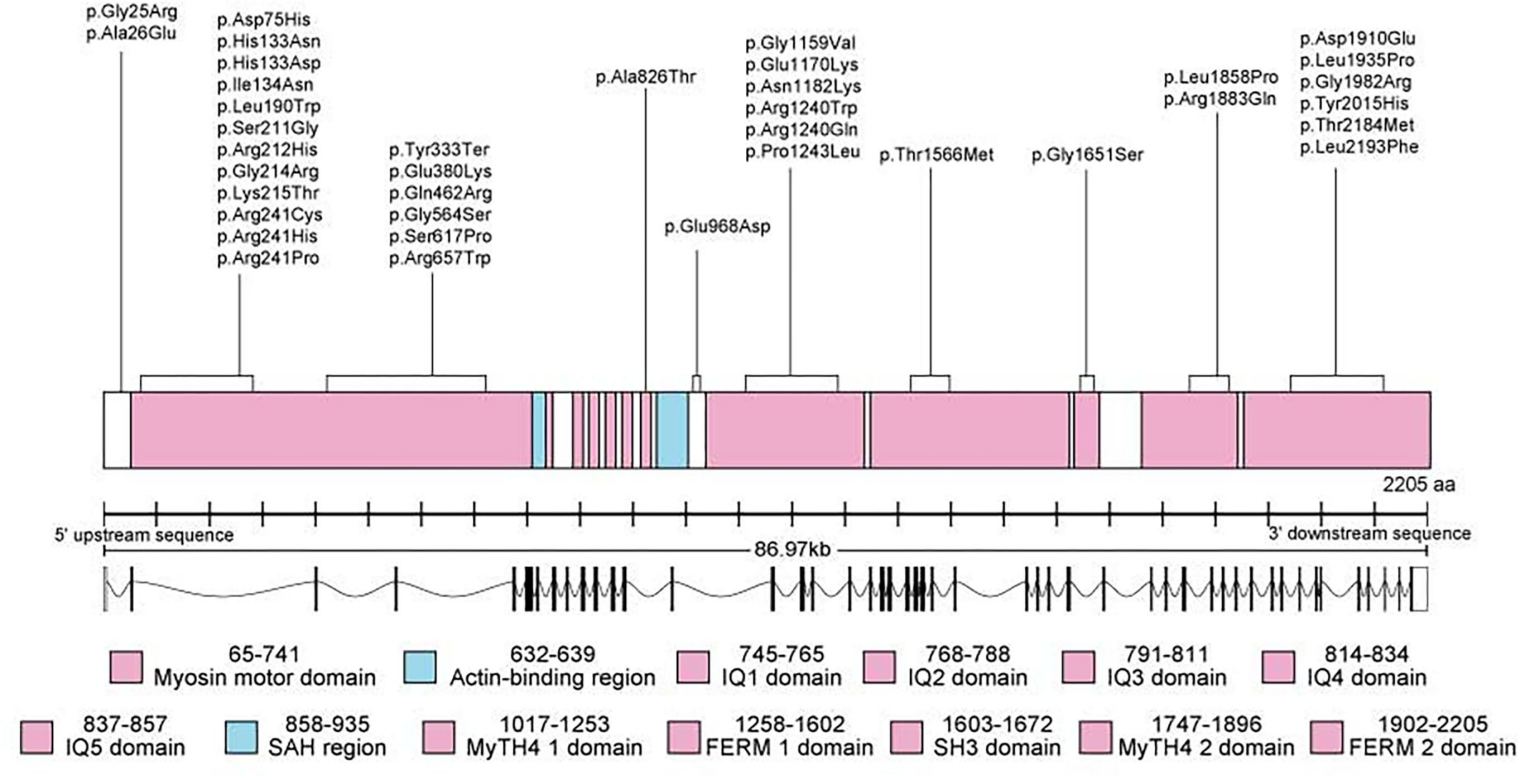
A schematic diagram of the genetic and protein structures of myosin-VIIa. The positions of detected *MYO7A* variants in our cohort. IQ1-5, 1-5 isoleucine-glutamine motifs; FERM, radixin-moesin; MyTH4, myosin tail homology 4; SAH, stable single a-helix; SH3, SRC homology 3.

Pattern ERG P50 showed a high degree of inter-ocular symmetry (slope = 0.97; *r*^2^ = 0.95). Pattern ERG P50 was undetectable in four of five of those with the most severe ffERG reductions, but in others amplitudes were between 50% to 89% (*N* = 8) or 90% to 100% (*n* = 3) of the lower limit of normal or were well within the reference range (*n* = 4). There was a statistically significant correlation between PERG50 and BCVA (*P* ≤ 0.005; *n* = 19). There was no clear relationship between age and pattern ERG P50 reduction. Cases 16–19 (Fig. 6) had the mildest ERG phenotypes without LA 30Hz ERG delay, and all four had fundus changes localized to the inferior retina, in keeping with restricted disease and a clinical diagnosis of sector RP (Fig. 4). Subject 15 (Fig. 6) had retinal changes that extended over three quadrants of the posterior pole, and showed marked flicker ERG delay, more in keeping with generalized retinal disease.

Of the 19 subjects that underwent ISCEV Standard testing the only two to be nullizygous were also the youngest, and both had a severe ffERG phenotype (cases 5 and 9 in Figure 6, aged nine and 11 years). Of the five children tested with lower eyelid skin electrodes (ages seven to 11 years), four of five had double null *MYO7A* variants, and all five showed marked reductions in scotopic and photopic flash ERGs consistent with a severe photoreceptor dystrophy.

### Molecular Genetics

Eighty-three disease-causing variants were identified, of which 35 (42.2%) were missense, 18 (21.7%) were frameshift variants, 14 (16.8%) were stop-gain variants, seven (8.4%) were splice-acceptor variants, seven (8.4%) were splice-donor variants, one (1.2%) was an in-frame deletion, and one was gross deletion (1.2%) (Fig. 7). To the best of our knowledge, 18 variants reported herein were novel (see Supplementary Table S2). The most frequent variants were (1) c.133-2A>G, (2) c.3719G>A, p.(Arg1240Gln), and (3) c.5886_5888del, p.(Phe1963del). All were present in five alleles of unrelated families.

Fifty-three patients (66.2%) had NDN genotypes, and 27 patients (33.8%) had DN (nonsense, frameshift, splicing, or exon deletion) genotypes. Truncation variants escaping nonsense-mediated mRNA decay were categorized into NDN genotype as shown in the Supplementary Table S2.

Patients with an NDN phenotype had a wider EZW and hyperAF ring area at baseline compared to DN baseline phenotype (5176.8 µm and 42.8 mm^2^ vs. 4444.8 µm and 20.5 mm^2^, respectively). ANOVA showed a not statistically significant difference in the hyperAF ring area adjusted between the different groups of age. On the other hand, EZW showed a statistically significant difference in the group of age above 40 years. In addition, there were no statistically significant differences between NDN and DN genotypes compared across the different groups of age with respect to BCVA at baseline.

Regarding the sector RP patients (*n* = 6), all had NDN genotypes; four had missense variants, two had a combination of a missense with a null variant (one frameshift and one nonsense). Two of the sector patients (MYO7A 013 and 014) are siblings. Regarding the six mild cases, five had NDN genotypes (two cases with two missense variants, three had a combination of one missense and one null variant), and one case was DN. Among these 12 cases, 10 variants within this group (nine missense and one nonsense) were not found in the rest of the cohort. Five patients from these two groups (sector and mild; Supplementary Table S1) had milder hearing impairment compared to the rest of the cohort who had SNHL (MYO7A 013, 014, 030, 038, and 040).

## Discussion

This retrospective observational study represents the largest cohort to date of patients with molecularly confirmed USH1 associated with *MYO7A*. The clinical phenotype, imaging and electrophysiological features, and longitudinal evaluation are detailed; providing novel findings that improve genetic counseling, advice on prognosis, and have implications for clinical trial design and participant stratification. Eighteen novel *MYO7A* variants are reported.

Variants in the *MYO7A* and *USH2A* genes are the most common causes of USH, respectively, representing more than 50% of USH1 and 70% of USH2. USH1 presents with early-onset RP, congenital severe SNHL and balance dysfunction.^9^^,^^29^^,^^30^ In our cohort, the mean age of onset of visual symptoms was 12.1 years old, which is consistent with previous reports (nine to 13 years).^31^^–^^33^ The mean age at the first visit was 25.4 years, and the BCVA was 0.4 LogMAR. Different cohorts have reported similar or slightly worse BCVA at the time of their first visit, 0.39 - 0.6 LogMAR.^31^^,^^34^^,^^35^ Regarding progression, the mean follow-up was 16.2 ± 11.4 years and the change in BCVA over time was 0.02 LogMAR/per year. Khateb et al.^31^ (*n* = 53) reported a rate of progression of 0.025 LogMAR/year. A previous study from Moorfields Eye Hospital (Lenassi et al.^34^) reported the follow-up of up-to three years of 20 patients with USH1 associated with *MYO7A,* where the median BCVA was 0.4 LogMAR, thereby in keeping with our larger cohort and a rate of yearly decline of 1.86%.

The EZW and hyperAF ring area of our cohort was largely symmetric between eyes, as indicated by Bland-Altman plots. One case had asymmetric EZW at both the baseline and follow-up timepoints (five years old; MYO7A 075), whereas another case had asymmetric EZW at baseline only, caused by a macular hole (MYO7A 002). Only one case had asymmetric hyperAF rings between eyes, due to peripapillary atrophy in one eye (MYO7A 046).

Seventy-eight percent of the patients who had fundus images available had a variable degree of BSL pigmentation, and only nine (14.1%) patients had no pigment. This contrasts with a French cohort where they found only 26% of the patients had BSL pigment.^31^ Patients with atypical milder phenotypes and sector RP have been described in the literature in association with *MYO7A*.^31^^,^^35^^–^^37^

In our cohort, six patients (7.5%) had sector RP, two of whom have previously been reported by Georgiou et al.^38^ In addition, six other patients (7.5%) had a milder generalized phenotype characterized by a large hyperAF ring and a greater central area of preserved retina until a later age. The BCVA was significantly better in these two groups compared (*n* = 12) to the patients outside these subgroups (*P* ≤ 0.001). An NDN genotype was found in 10 of these 12 patients.

Our cohort had a similar proportion of patients with sector RP (7.5%) compared with a recently published cohort of patients with USH1 associated with *CDH23* (9.5%).^39^^,^^40^ In this subgroup of patients (mild and sector), we found 10 variants not present in the rest of the cohort, shown in Supplementary Table S1. These alleles might represent hypomorphic variants that, when associated with a severe allele, give rise to this milder/restricted phenotype; however, functional studies would be required to classify these variants adequately.

Interestingly, patients MYO7A 069 (mild phenotype), 074, and 078 (typical phenotype) had the same genotype (c.133-2A>G; c.3719G>A, p.Arg1240Gln). Patient 069 had a larger ring of increased FAF signal and a better BCVA at the age of 51 years, compared to subjects 74 and 78 who were younger and have worse BCVA and a more severe RP phenotype. The variant c.133-2A>G was associated with a milder phenotype in a previous report.^31^

The measurement of the hyperAF ring, when present, has become a useful prognostic variable in research and clinical practice for patients with RP. This is because it has been shown through functional and imaging studies that the inner border of the ring delineates the limits of a relatively intact EZ, and a normal retina lies within the ring.^41^^–^^45^ Previous studies in a cohort of combined USH1 and USH2 patients have reported a progression of 4% per year^46^ and in *MYO7A* 2.54%.^31^ In our study, we found a change over time of the hyperAF ring area of 1.2 mm^2^ per year. Recent prospective studies published by Testa et al.^47^ found that a hyperAF ring was present in 46% of patients with *MYO7A*, similar to our study in which it was present in 50.7% of the patients with FAF available.

It is not uncommon for patients with RP to have complications that can be potentially treatable such as cataract and CMO. The prevalence of ERM has been previously reported to be higher for *MYO7A* than other forms of RP, up to 56%.^31^ However, in this study, only 22% of the patients had unilateral or bilateral ERM, similar to previous reports (22–27%) by Triolo et al.^48^ and Liew et al.^49^ Twenty-four of our patients had CMO in at least one eye, showing a similar prevalence compared to previous reports in patients with *MYO7A* (27%–58%).^46^^,^^47^^,^^49^ Sixty percent of our patients developed cataracts; this is higher than the average reported by previous studies (23%–29%)^31^^,^^34^^,^^47^^,^^49^ and can potentially be attributed to the longer average follow-up 16.2 ± 11.4 years in our cohort.

The horizontal EZW is a measurement of central retinal structural preservation that correlates with the hyperAF ring boundary.^50^ The rate of EZW loss in a smaller French cohort has been reported as higher (4%)^31^ than our cohort, where the mean loss of EZW was 25.2 µm per year (1.1% per year). More advanced imaging technologies, such as adaptive optics (AO), allow direct visualization of photoreceptor integrity, with AO studies illustrating that the cone density at the fovea can be decreased before structural changes are evident on OCT.^51^^,^^52^

We explored genotype-phenotype associations by comparing the EZW and hyperAF ring area between NDN and DN genotype groups. We found that both anatomical parameters were better preserved in the NDN group. Showing a statistically significant difference in the group of age above 40 years (*P* = 0.008) However, it failed to show statistical significance in the rest of the subgroups of age in both variables. This could be associated with uneven distribution of the subgroups of patients that had follow-up after being stratified for analysis, and the presence of some of the mild cases that have a greater preservation at older age (Fig. 1).

Only 38% of the patients in this study had a cochlear implant despite the diagnosis of congenital SNHL. Out of the six patients with sector RP, five had moderate hearing loss with sufficient hearing to develop speech with the help of hearing aids. In comparison, five patients from the mild phenotype group had profound SNHL and only one had moderate hearing loss. All patients with mild hearing loss harbored an NDN phenotype, with two missense variants. Recent systematic reviews have shown that providing cochlear implants in early childhood can lead to better spoken language acquisition and speech recognition.^53^^,^^54^ This is supported by a recent study by Karltorp et al.^55^ that showed that language development in children with a cochlear implant before nine months of age was similar to the normative data of four-year-old children.

In the current study, ERGs revealed a wide range of rod and cone photoreceptor dysfunction (from undetectable to normal), including most with a similar degree of rod and cone involvement. This is unlike classic cases of rod-cone dystrophy, as seen in many cases of mild-moderate isolated RP. There was also pattern ERG evidence of relative or complete sparing of macular function in the majority. Only six of 24 who underwent ERG testing were nullizygous for *MYO7A* variants, but it is notable that these included six of the seven youngest children, all with ERG evidence of severe photoreceptor dysfunction. NDN genotypes were associated with a wider spectrum of ERG findings, including some severe and mild cases, but it is tempting to speculate that nullizygosity may be associated with more consistently earlier and severe retinal dysfunction, although investigations of larger *MYO7A* cohorts will be needed to better establish genotype-phenotype ERG correlations.

### Limitations

The retrospective nature of this study is associated with certain inherent limitations, such as variability in the available data, the lack of standardized protocol used for assessments, lack of axial length measurements to correct EZW measurements, and variable follow-up intervals. This could influence our analysis because the progression could depend on the stage of the disease. In this study, the change over time was calculated only with two measurements (baseline and follow-up), and although it has been described that the EZW and HyperAF ring do not necessarily follow a linear progression, we believe that our work will be useful for future natural history studies and clinical trials.^28^ The visual field data from our cohort was excluded from the analysis, which is known to be a useful functional assessment, because the information in the clinical records was minimal and from different platforms. Different genetic testing protocols were applied.

## Conclusions

This represents the largest and longest follow-up longitudinal cohort of molecularly confirmed patients with *MYO7A*-associated USH1. We described the clinical phenotype, detailed imaging, electrophysiological features, and progression, of a retrospective cohort of patients, expanding the description of functional and structural phenotypes and genetic variants.

*MYO7A-*associated USH1 appears to have a wide window for therapeutic intervention given the majority of patients retain central vision/architecture until the fifth decade of life. Also, given that SNHL in most cases precedes retinal involvement, these patients can be diagnosed early. Prospective natural history studies are needed to further determine clinical outcomes.

## Supplementary Material

Supplement 1

Supplement 2

Supplement 3

Supplement 4
