# Supplementary material for: Detailed Clinical, Ophthalmic, and Genetic Characterization of MYO7A-Associated Usher Syndrome
Source: Invest Ophthalmol Vis Sci. 2025 Apr 21;66(4):60. doi: 10.1167/iovs.66.4.60 (PMC12020961; doi:10.1167/iovs.66.4.60)
Supplement: Supplement 1 [file iovs-66-4-60_s001.pdf]

Supplementary Methods

In silico molecular genetic analyses

In silico molecular genetic analyses were performed for the detected *MYO7A* variants. Minor allele frequency for the identified variants in the general population was assessed in the Genome Aggregation Database (gnomAD) datasets. The population data and general coverage by whole exome sequence were also provided with the gnomAD database. General prediction scores were further calculated using MutationTaster, FATHMM, CADD, and REVEL. Functional prediction was performed employing SIFT, PROVEAN, and Polyphen 2. Human splicing finder 3.0 was applied for splicing defects prediction. Mammalian (PhyloP30way and PhastCons30way) and vertebrate (PhyloP100way and PhastCons100way) conservation were also investigated. The previously reported variants were surveyed with the HGMD database and ClinVar database (accessed in August 2024).

The pathogenicity of each detected variant was confirmed according to the American College of Medical Genetics and Genomics (ACMG) guidelines. The evolutionary conservation was assessed by multiple alignments of species of the *MYO7A* gene sequence using the Clustal Omega program (https://www.ebi.ac.uk/Tools/msa/clustalo/)

1. Lefter M, Vis JK, Vermaat M, den Dunnen JT, Taschner PEM, Laros JFJ. Mutalyzer 2: next-generation HGVS nomenclature checker. *Bioinformatics*. 2021;37(18):2811-2817. doi:10.1093/bioinformatics/btab051

2. Richards S, Aziz N, Bale S, et al. Standards and guidelines for the interpretation of sequence variants: a joint consensus recommendation of the American College of Medical Genetics and Genomics and the Association for Molecular Pathology. *Genet Med*. 2015;17(5):405-424. doi:10.1038/gim.2015.30

3. Abou Tayoun AN, Pesaran T, DiStefano MT, et al. Recommendations for interpreting the loss of function PVS1 ACMG/AMP variant criterion. *Hum Mutat*. 2018;39(11):1517-1524. doi:10.1002/humu.23626
