# Supplementary material for: Detailed Clinical, Ophthalmic, and Genetic Characterization of MYO7A-Associated Usher Syndrome
Source: Invest Ophthalmol Vis Sci. 2025 Apr 21;66(4):60. doi: 10.1167/iovs.66.4.60 (PMC12020961; doi:10.1167/iovs.66.4.60)
Supplement: Supplement 4 [file iovs-66-4-60_s004.pdf]

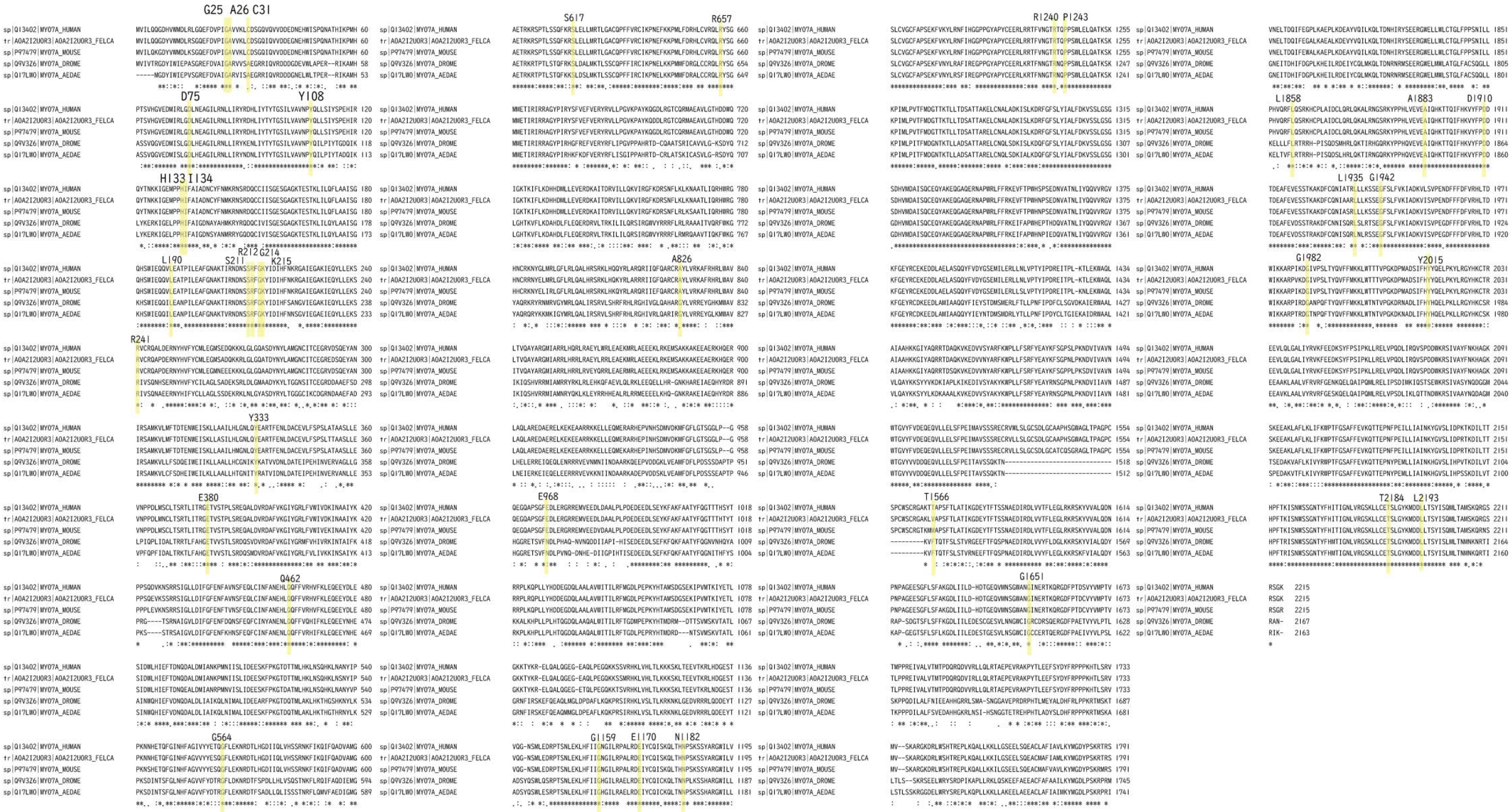

Supplementary Table 3:Evoluationary conservation of novel mutations identified in our study.
